# Metformin Impairs Linsitinib Anti-Tumor Effect on Ovarian Cancer Cell Lines

**DOI:** 10.3390/ijms252211935

**Published:** 2024-11-06

**Authors:** Diana Luísa Almeida-Nunes, João P. N. Silva, Mariana Nunes, Patrícia M. A. Silva, Ricardo Silvestre, Ricardo Jorge Dinis-Oliveira, Hassan Bousbaa, Sara Ricardo

**Affiliations:** 1 Associate Laboratory i4HB—Institute for Health and Bioeconomy, University Institute of Health Sciences—CESPU, 4585-116 Gandra, Portugal; nunesdiana@msn.com (D.L.A.-N.); patricia.silva@cespu.pt (P.M.A.S.); ricardo.dinis@iucs.cespu.pt (R.J.D.-O.); 2 UCIBIO—Applied Molecular Biosciences Unit, Toxicologic Pathology Research Laboratory, University Institute of Health Sciences (1H-TOXRUN, IUCS-CESPU), 4585-116 Gandra, Portugal; 3 Differentiation and Cancer Group, Institute for Research and Innovation in Health (i3S) of the University of Porto, 4200-135 Porto, Portugal; mnunes@i3s.up.pt; 4 UNIPRO—Oral Pathology and Rehabilitation Research Unit, Institute of Health Sciences (IUCS), Cooperativa de Ensino Superior Politécnico e Universitário (CESPU), Rua Central de Gandra 1317, 4585-116 Gandra, Portugal; joaosilva_06@hotmail.com (J.P.N.S.); hassan.bousbaa@iucs.cespu.pt (H.B.); 5 School of Medicine and Biomedical Sciences (ICBAS), University of Porto, 4050-313 Porto, Portugal; 6 UCIBIO—Applied Molecular Biosciences Unit, Translational Toxicology Research Laboratory, University Institute of Health Sciences (1H-TOXRUN, IUCS-CESPU), 4585-116 Gandra, Portugal; 7 Life and Health Sciences Research Institute (ICVS), School of Medicine from University of Minho, 4710-057 Braga, Portugal; ricardosilvestre@med.uminho.pt; 8 ICVS/3B’s—PT Government Associate Laboratory, 4710-057 Braga/Guimarães, Portugal; 9 Department of Public Health and Forensic Sciences, and Medical Education, Faculty of Medicine from University of Porto (FMUP), 4050-319 Porto, Portugal; 10 FOREN—Forensic Science Experts, 1400-136 Lisboa, Portugal

**Keywords:** ovarian cancer, linsitinib, IGF-1 signaling pathway, metformin, cell viability, apoptosis

## Abstract

Ovarian cancer (OC) remains one of the leading causes of cancer-related mortality among women. Targeting the insulin-like growth factor 1 (IGF-1) signaling pathway has emerged as a promising therapeutic strategy. Linsitinib, an IGF-1 receptor (IGF-1R) inhibitor, has shown potential in disrupting this pathway. Additionally, metformin, commonly used in the treatment of type 2 diabetes, has been studied for its anti-cancer properties due to its ability to inhibit metabolic pathways that intersect with IGF-1 signaling, making it a candidate for combination therapy in cancer treatments. This study explores the anti-cancer effects of linsitinib and metformin on OVCAR3 cells by the suppression of the IGF-1 signaling pathway by siRNA-mediated *IGF-1* gene silencing. The goal is to evaluate their efficacy as therapeutic agents and to emphasize the critical role of this pathway in OC cell proliferation. Cellular viability was evaluated by resazurin-based assay, and apoptosis was assessed by flux cytometry. The results of this study indicate that the combination of linsitinib and metformin exhibits an antagonistic effect (obtained by SynergyFinder 2.0 Software), reducing their anti-neoplastic efficacy in OC cell lines. Statistical analyses were performed using ordinary one-way or two-way ANOVA, followed by Tukey’s or Šídák’s multiple comparison tests. While linsitinib shows promise as a therapeutic option for OC, further research is needed to identify agents that could synergize with it to enhance its therapeutic efficacy, like the combination with standard chemotherapy in OC (carboplatin and paclitaxel).

## 1. Introduction

Ovarian cancer (OC) is the second most common malignant gynecologic neoplasm in Western countries and has the highest mortality rate of all gynecological tumors due to its late diagnosis [1]. Advanced OC is characterized by metastases in the peritoneal cavity and/or in the retroperitoneal lymph nodes, supporting extensive disease spread beyond the abdomen. Metastasis from the primary ovarian/fallopian tube tumors can occur through three routes, transcoelomic, lymphatic, or hematogenous, with the first being the most common. Peritoneal carcinomatosis and excess peritoneal fluid known as malignant ascites occur in 90% of patients with advanced OC [2]. According to the World Health Organization, the majority of OCs (90% of cases) are of epithelial origin and can be classified into five main types based on histopathology, immune profile, and molecular analysis: high-grade serous carcinoma (HGSC, 70%), endometrioid carcinoma (EC, 10%), clear-cell carcinoma (CCC, 6–10%), low-grade serous carcinoma (LGSC, 5%), and mucinous carcinoma (MC, 3–4%) [1]. Genomic mutations have an important role in the pathogenesis of OC. High-prevalence somatic (non-germline) mutations (>5%) have been reported in a limited number of genes in OC. The genes whose functional perturbation likely contributes to ovarian carcinogenesis include *TP53*, *CTNNB1*, *PTEN*, *KRAS*, *PIK3CA*, and *AKT1.* Hereditary (germline) mutations in *BRCA1* and *BRCA2* are associated with epithelial OCs that typically occur at an earlier age. In contrast, sporadic tumors are more frequently HGSC with mutations in the *TP53* gene [3]. Over recent decades, taxane- and platinum-based chemotherapy has been the standard of care for OC patients and, only in the previous year [4,5], targeted therapy such as anti-vascular endothelial growth factor receptor (VEGFR) and poly ADP ribose polymerase (PARP) inhibitors were included in the management of this type of cancer [6]. Despite efforts, the survival rate has not significantly improved, highlighting the need for new therapeutic strategies.

**Figure 1:**
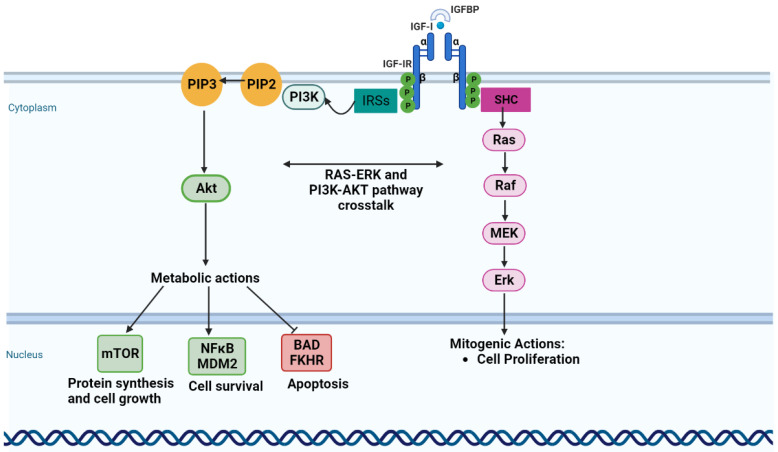
The insulin-like growth factor 1 signaling pathway. Insulin-like growth factor 1 (IGF-1) activates both phosphatidylinositol 3-kinase/Akt and Ras/mitogen-activated protein kinase pathways, resulting in cell proliferation, increased protein synthesis, and cell growth. Phosphatidylinositol 3-kinase/Akt activates nuclear factor-κB and MDM2 for cell survival and inhibits apoptosis through inhibition of BAD and FKHR. Akt—Ak strain transforming; BAD—BCL2-associated agonist of cell death; Erk—extracellular-signal-regulated kinase; FKHR—Forkhead transcription factor FOXO1; IGF-I—insulin-like growth factor 1; IGF-IR—insulin-like growth factor 1 receptor; IGFBP—insulin-like growth factor binding protein; IRSs—insulin receptor substrate proteins; MDM2—mouse double minute 2; MEK—mitogen-activated protein kinase; mTOR—mammalian target of rapamycin; NFκB—nuclear factor immunoglobulin κ chain enhancer-B cell; P—phosphate; PI3K—phosphatidylinositol 3-kinase; PIP2—phosphatidylinositol 3, 4 phosphates; PIP3—phosphatidylinositol 3, 4, 5 phosphates; Raf—rapidly accelerated fibrosarcoma; Ras—rat sarcoma; SHC—Src homology/collagen. Figure created in BioRender.com.

The insulin-like growth factor 1 (IGF-1) signaling pathway is a potential therapeutic target since it is crucial in the development, maintenance, progression, survival, and chemotherapeutic response in OC [5,7]. IGF-1 and its receptor insulin-like growth factor 1 receptor (IGF-1R) are overexpressed in serous ovarian carcinoma [8]; the activation of this signaling pathway enhances the proliferation and tumorigenicity in OC cell lines [9] and is shown to be associated with chemoresistance [10,11]. Downstream effectors of the insulin growth factor (IGF) pathway, such as the phosphatidylinositol 3-kinase (PI3K)-Ak strain transforming (Akt)/mammalian target of rapamycin (mTOR) pathway and rapidly accelerated fibrosarcoma (RAF)/mitogen-activated protein (MAP) kinase (Figure 1), play well-established roles as mitogens in carcinogenesis [12,13]. Several ongoing clinical trials are exploring the efficacy of IGF-1R inhibitors in OC [14]. Linsitinib (OSI-906) is one of these inhibitors, and it targets the tyrosine kinase domain of both IGF-1R and insulin-receptor (IR) [8], preventing tumor cell proliferation and inducing apoptosis [15]. Additionally, linsitinib has been shown to restore the sensitivity of ovarian clear-cell carcinoma cells to cisplatin by silencing the IGF-1R/Akt signaling pathway [16].

Metformin, a biguanide drug, was first discovered in the 1920s as an anti-diabetic medication and has been improving mortality rates in several other conditions, such as cancer, obesity, polycystic ovary syndrome, and metabolic syndrome [17]. Regarding cancer, metformin has shown significant survival benefits in colorectal and prostate cancers [18] and in hormone-receptor-positive breast tumors [19]. Despite some clinical trials demonstrating an association between metformin and improved prognosis in cancer patients, this association has not been fully established in OC [20]. The anti-diabetic effect of metformin is mediated through the enzyme adenosine monophosphate-activated protein kinase (AMPK). Metformin reduces hepatic gluconeogenesis and promotes glucose uptake by muscles via AMPK activation, thus lowering blood glucose and insulin levels and reducing insulin resistance, which is a risk factor for OC [21,22]. The anti-neoplastic effect of metformin in OC involves the activation of AMPK and various other mechanisms, such as the IGF-1 pathway, since IGF-1R is overexpressed in OC [23]. Metformin treatment lowers the secretion of IGF-1 [24], downregulates the expression of IR [25] and IGF-1R [24,26], inhibits the phosphorylation of IGF-1R [25], and increases the expression of insulin-like growth factor binding protein 1 (IGFBP-1) [26]. This leads to the inhibition of the PI3K/Akt [24] and extracellular-signal-regulated kinase (ERK)1/2 signaling pathways [27], resulting in the inhibition of cell growth and proliferation, the decreased synthesis of proteins and fatty acids, and the reduced paracrine and endocrine release of pro-proliferative systemic factors [28]. In addition to IGF-1-related signaling pathways, metformin also affects the activity of the transcription factor signal transducer and activator of transcription 3 (STAT3), which is usually activated by various growth factors and cytokines, leading to its dimerization, translocation to the nucleus, and induction of multiple transcription pro-survival and pro-proliferative genes [28]. STAT3 levels are elevated in endometrial cancer cells, particularly the serine-phosphorylated form, phospho-STAT3 Ser727 [29]. High glucose concentrations induce the transcription of STAT3 and its upstream regulators Janus kinases 1 and 2 (JAK1/2), while metformin treatment reduces total STAT3 protein and phospho-STAT3 Ser727 [30]. This is associated with a significantly decreased expression of multiple pro-survival downstream targets of STAT3, including c-Myc and B-cell lymphoma (Bcl)-2 and –XL, providing another possible mechanism for metformin’s anti-cancer activity [30].

In this study, we aimed to investigate the effect of inhibiting the IGF-1 signaling pathway in an IGF-1-overexpressing OC cell line by siRNA-mediated *IGF-1* gene silencing. We assessed the impact on OC cell proliferation, both in untreated and treated cells with metformin and linsitinib, to demonstrate the role of this pathway in regulating OC cell proliferation. This study aims to highlight the importance of the IGF-1 signaling pathway in the proliferation of OC cell lines and to evaluate the interaction between metformin and linsitinib, since both target this specific pathway.

## 2. Results

### 2.1. IGF-1 Is Overexpressed in Ovarian Cancer Cell Lines

**Figure 2:**
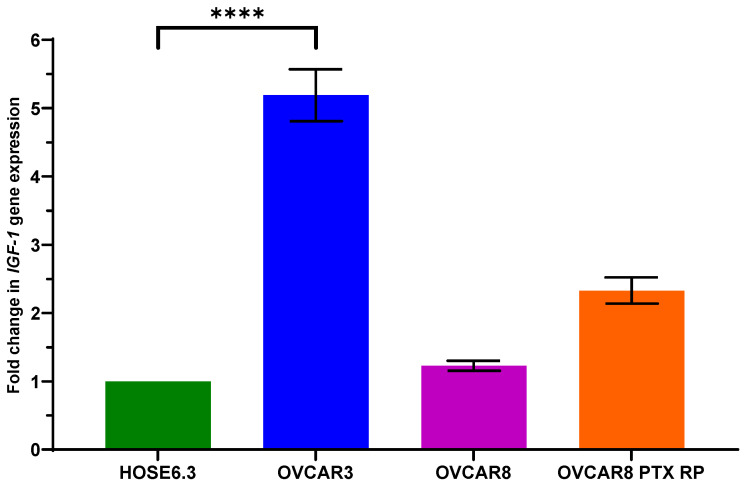
*IGF-1* gene expression in ovarian cell lines. Bar chart showing relative IGF-1 mRNA expression levels in HOSE6.3, OVCAR3, OVCAR8, and OVCAR8 PTX R P cell lines determined by qRT-PCR with β-Actin and GAPDH used as housekeeping genes. The assays were carried out in triplicate in at least three independent experiments. Data are expressed as mean ± standard error of mean deviation (SEM) and plotted using GraphPad Prism Software Inc., San Diego, CA, USA v9. Statistical analysis was performed using ordinary one-way ANOVA followed by Šídák’s multiple comparison test, and values of **** < 0.0001 were considered statistically significant.

To select the best cell line model to evaluate the effect of IGF-1 signaling pathway inhibition, we evaluated IGF-1 mRNA expression levels in HOSE6.3, OVCAR3, OVCAR8, and OVCAR8 PTX R P using qRT-PCR. The results showed that IGF-1 mRNA levels were overexpressed in all OC cell lines (OVCAR3, OVCAR8, and OVCAR8 PTX R P) under study compared to the non-tumor ovarian cell line, HOSE6.3 (Figure 2). The most significant difference was obtained with OVCAR3 (*p*-value < 0.0001), where IGF-1 mRNA levels were increased five-fold; therefore, this OC cell line model was chosen for subsequent assays.

### 2.2. IGF-1 Signaling Pathway Inhibition Leads to Increased Cell Death in OVCAR3

**Figure 3:**
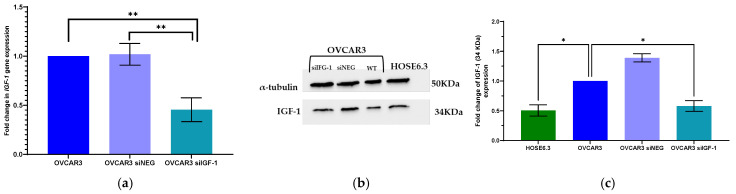
Silencing of *IGF-1* gene in OVCAR3 cell line. (**a**) Bar chart showing relative IGF-1 mRNA expression levels in OVCAR3, OVCAR3 transfected with siRNA control (OVCAR3 siNEG), and OVCAR3 transfected with siRNA of IGF-1 (OVCAR3 siIGF-1) determined by qRT-PCR. β-Actin and GAPDH were used as housekeeping genes. (**b**) Representative Western blot showing IGF-1 protein expression in HOSE6.3, OVCAR3, OVCAR3 siNEG, and OVCAR3 siIGF-1 cell lines. α-tubulin was used as a loading control. (**c**) Bar chart showing relative IGF-1 protein expression levels in HOSE6.3, OVCAR3, OVCAR3 siNEG, and OVCAR3 siIGF-1 determined by ImageJ 1.4v software. α-tubulin intensity levels were used as a control. The assays were carried out in triplicate in at least three independent experiments. Data are expressed as mean ± standard error of mean deviation (SEM) and plotted using GraphPad Prism Software Inc. v9. Statistical analysis was performed using ordinary one-way ANOVA followed by Šídák’s multiple comparison test and values of * < 0.05 and ** < 0.001 were considered statistically significant.

To better understand the effect of the IGF-1 pathway on OVCAR3 cells, they were transfected with siRNA targeting the *IGF-1* gene, and cell proliferation and cell death were evaluated. We obtained an efficiency of up to 60% in IGF-1 silencing at mRNA and protein levels (Figure 3). Interestingly, Western blot analysis showed increased IGF-1 protein levels in OVCAR3 compared to non-tumor cells (Figure 3c), confirming the overexpression previously observed at the mRNA level (Figure 2).

**Figure 4:**
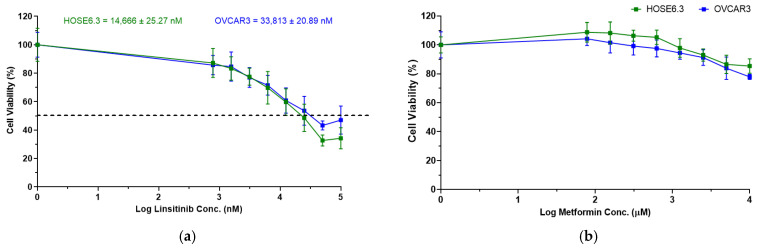
Dose–response curves for HOSE6.3 and OVCAR3 of drugs linsitinib and metformin. (**a**) Dose–response curves for HOSE6.3 and OVCAR3 cells were obtained by Presto Blue assay after exposure to increasing concentrations of linsitinib (780 to 100,000 nM) for 48 h. (**b**) Dose–response curves for HOSE6.3 and OVCAR3 cells were obtained by Presto Blue assay after exposure to increasing concentrations of metformin (80 to 10,000 μM) for 48 h. IC_50_ values are represented by a dotted line in each dose–response curve and are mentioned below. The assays were carried out in triplicate in at least three independent experiments. Data are expressed as mean ± standard error of mean deviation (SEM) and plotted using GraphPad Prism Software Inc. v9.

Next, we analyzed the anti-tumor potential of linsitinib and metformin as a single agent on HOSE6.3 and OVCAR3 cells using increasing concentrations (see Section 4.6—Drugs Treatment) after 48 h of treatment exposure, allowing to obtain a dose–response curve to calculate IC_50_ values for linsitinib, which was further used in the combination studies. These results showed that linsitinib displayed an anti-tumor activity in OVCAR3 cells at IC_50_ 33,813 ± 20.89 nM, but, unfortunately, at a lower IC_50_ value for non-tumoral cells (HOSE6.3) (14,666 ± 25.27 nM) (Figure 4a). In addition, even though metformin showed a cytotoxic effect, we did not reach the IC_50_ values at the highest concentration tested (10,000 μM) (Figure 4b). All experiments showed no differences between control cells with/without the vehicle.

**Figure 5:**
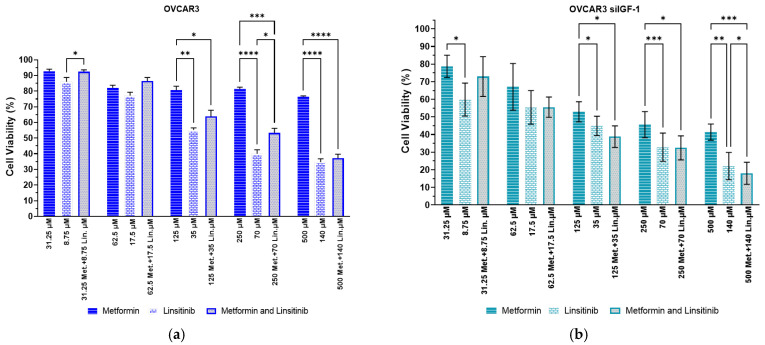
Linsitinib demonstrates high efficacy in reducing the cellular viability of OVCAR3 and OVCAR3 siIGF-1. (**a**) Bar charts showing cell viability of OVCAR3 cells obtained by Presto Blue assay after exposure to a fixed-dose ratio of linsitinib combined with metformin. (**b**) Bar charts showing cell viability of OVCAR3 siIGF-1 cells obtained by Presto Blue assay after exposure to a fixed-dose ratio of linsitinib combined with metformin. All assays were performed in triplicate in at least three independent experiments. Data are expressed as mean ± standard deviation and plotted using GraphPad Prism Software Inc. v9. Statistical analysis was performed using ordinary two-way ANOVA followed by Šidák’s multiple comparison test, and values of * < 0.05, ** < 0.001, *** < 0.005, and **** < 0.0001 were considered statistically significant.

After obtaining the IC_50_ values for linsitinib, the effect of its combination with metformin was evaluated using the combination model previously described [31]. Briefly, OVCAR3 and OVCAR3 siIGF-1 cells were exposed to linsitinib and metformin as single agents and tested in combination with a fixed-dose ratio of each drug. The single drug tests with linsitinib and metformin showed that viability was more reduced in OVCAR3 siIGF-1 than in OVCAR3 (Figure 5). In OVCAR3 cells, the combination of linsitinib and metformin (250 µM metformin + 70 µM linsitinib) had less anti-tumoral activity than linsitinib treatment alone (70 µM linsitinib), with *p*-value < 0.05 (Figure 5a). Furthermore, OVCAR3 siIGF-1 cells had a more significant decrease in cellular viability with linsitinib alone than with metformin alone or with the combination of metformin and linsitinib, except for the last condition of both drugs combined (500 µM metformin + 140 µM linsitinib) where we observed that the combination has more anti-tumoral activity than the linsitinib alone (140 µM), with *p*-value < 0.05 (Figure 5b). In summary, metformin alone has an insufficient effect on the viability of both cell lines.

**Figure 6:**
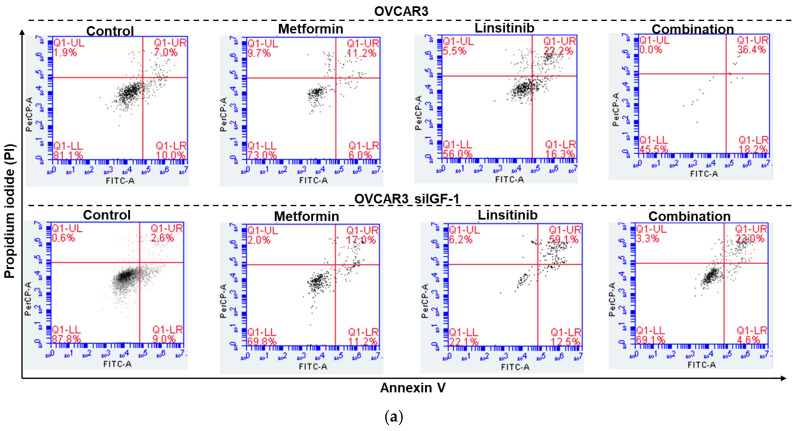

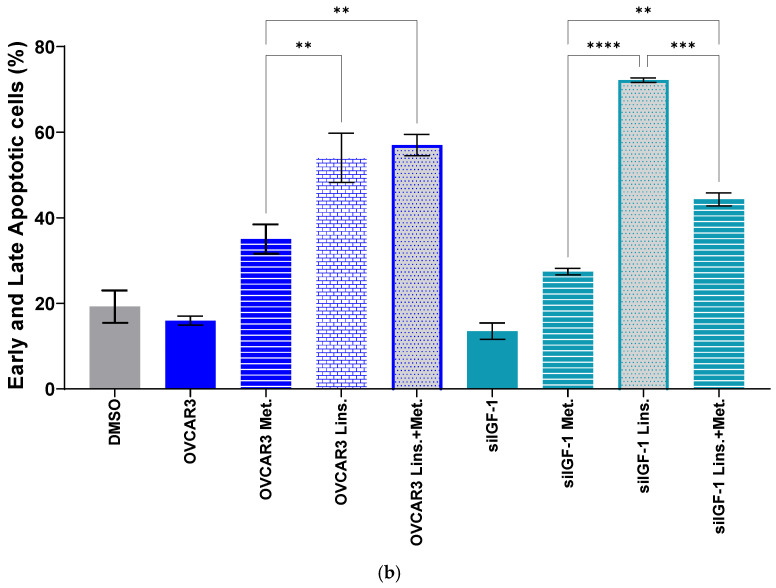
Stain with Annexin V/PI and analyzed by flow cytometry to confirm the cellular viability using the drugs linsitinib and metformin in the OVCAR3 and OVCAR3 siIGF-1 cells. (**a**) Representative flow cytometry histogram of propidium iodide (PI) versus annexin V (FITC-A) intensity in OVCAR3 and OVCAR3 siIGF-1 before (control–DMSO) and after exposure to metformin (500 µM), linsitinib (35 µM), and the combination of both drugs, during 48 h. DMSO was used as a control. The quadrants Q were defined as Q1 = live cells (Annexin V-negative/PI-negative), Q1-LR = early stage of apoptosis (Annexin V-positive/PI-negative), Q1-UL = late stage of apoptosis (Annexin V-positive/PI-positive), and Q1-UL = necrosis (Annexin V-negative/PI-positive). (**b**) Bar charts showing the percentage of Annexin V-positive cells (early and late stage of apoptosis) to the different conditions of OVCAR3 and OVCAR3 siIGF-1. The assays were carried out in triplicate in at least three independent experiments. Data are expressed as mean ± standard error of mean deviation (SEM) and plotted using GraphPad Prism Software Inc. v9. Statistical analysis was performed using ordinary one-way ANOVA followed by Šídák’s multiple comparison test and values of ** < 0.001, *** < 0.005, and **** < 0.0001 were considered statistically significant.

To confirm the previous results, OVCAR3 and OVCAR3 siIGF-1 cells treated for 48 h with linsitinib, metformin, and a combination of both were stained with Annexin V/PI and analyzed by flow cytometry (Figure 6a). In untreated OVCAR3 cells, the percentage of apoptosis was residual (15.99 ± 1.04%), while in OVCAR3 cells treated with linsitinib and with a combination of metformin and linsitinib, a significant increase in apoptotic cells (54.01 ± 5.74% and 57.02 ± 2.48%, respectively) was observed when compared to the cells treated alone with metformin (35.02 ± 3.44%), with *p*-value < 0.001 shown in Figure 6b. In the OVCAR3 cell line transfected with siRNA of IGF-1 (OVCAR3 siIGF-1), a significant increase in apoptotic cells was observed when treated with linsitinib (72.18 ± 0.55%) when compared to the cells treated only with metformin (27.42 ± 0.77%), with *p*-value < 0.0001. In these cells, we also observed a significant increase in apoptotic cells treated with the combination of both drugs (44.31 ± 1.52%) when compared to the cells treated with only metformin (27.42 ± 0.77%), with *p*-value < 0.001. However, it is less increased than when using only linsitinib (72.18 ± 0.55%), with *p*-value 0.005 as shown in Figure 6b. Thus, these results corroborate that linsitinib has more of an effect on cancer cell viability, especially in OVCAR3 siIGF-1, than metformin alone.

In accordance with the mentioned results, OVCAR3 and OVCAR3 siIGF-1 cells show morphological differences for linsitinib and combined treatments compared to the vehicle. OVCAR3 presents a more aggressive phenotype when treated with linsitinib, i.e., decreasing cell number, less aggregate formation, and smaller and rounded cells, indicative of cell death compared to vehicle and metformin treatment (see Supplementary Data, Figure S1). OVCAR3 siIGF-1 shows more cell death in linsitinib and combinatory treatments than with metformin treatment.

### 2.3. Combining Metformin and Linsitinib Has an Antagonist Effect on OVCAR3

**Figure 7:**
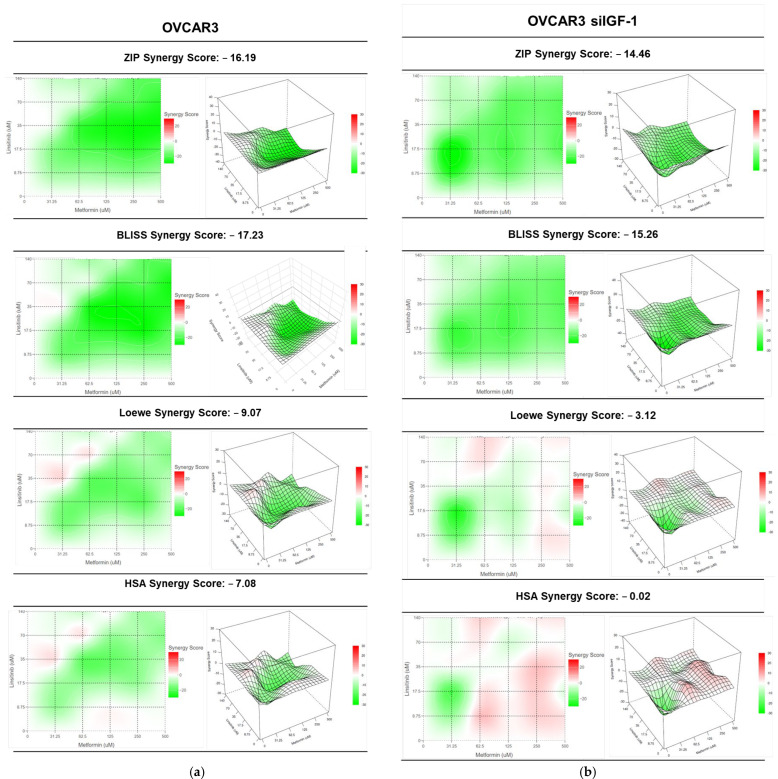
Combining linsitinib with metformin has an antagonist effect on OVCAR3 and OVCAR3 siIGF-1 cells. (**a**) ZIP, Bliss Independence, Loewe, and High Single Agent (HSA) synergy 2D and 3D plots showing drug antagonism of OVCAR3 cells after exposure to a fixed-dose ratio of linsitinib and metformin for 48 h. (**b**) ZIP, Bliss Independence, Loewe, and HSA synergy 2D and 3D plots showing drug antagonism of OVCAR3 siIGF-1 cells after exposure to a fixed-dose ratio of linsitinib and metformin for 48 h. The combined treatment was co-administered at the same time. All assays were performed in triplicate in at least three independent experiments. Synergy score: <10 (antagonism, green), =1 (additivity, white), and >10 (synergism, red).

Additionally, we investigated whether the two drugs could lead to synergistic effects. Since different methods for predicting synergism can result in different outcomes, we evaluated the drug interactions using the Zero-Interaction Potency (ZIP), Bliss Independence, Loewe, and High Single Agent (HSA) models (Figure 7) to compare results. These methods have different mathematical frameworks [32] and can produce slightly different results. The ZIP model captures the drug interaction relationships by comparing the change in the potency of the dose–response curves between individual drugs and their combinations [33]. The Bliss Independence model assumes a stochastic method in which two drugs produce their effects independently, and the expected combined effect can be evaluated based on the probability of these independent events occurring [34]. The Loewe additivity model defines the expected impact as if a drug were combined with itself [33]. The HSA method is one of the simplest synergy models and assumes that the expected combined effect equals the maximum of the single-drug responses at corresponding concentrations [34]. To perform these analyses, we used the SynergyFinder 2.0 Software, which allows for interactive analysis and visualization of multi-drug combination profiling data using four different synergism evaluation methods [35]. The synergy score for a drug combination is averaged over all the dose combination measurements, giving a positive (synergism) or negative (antagonism) value that could be observed in 2D and 3D synergy map dose regions, i.e., synergistic (red) and antagonistic (green) [35].

Even though different synergy evaluation models can produce varying results, our data indicate that the combination of linsitinib with metformin in OVCAR3 (Figure 7a) and OVCAR3 siIGF-1 (Figure 7b) demonstrated an antagonistic effect in all models used for this analysis. The ZIP model showed a synergy score of −16.19 and −14.46 for OVCAR3 and OVCAR3 siIGF-1, respectively. The Bliss model indicated a synergy score of −17.23 and −15.26 for OVCAR3 and OVCAR3 siIGF-1, respectively. The Loewe model indicated a synergy score of −9.07 and −3.12 for OVCAR3 and OVCAR3 siIGF-1, respectively. The HSA model showed a synergy score of −7.08 and −0.02 for OVCAR3 and OVCAR3 siIGF-1, respectively. Nonetheless, in Loewe and HSA models of the OVCAR3 siIGF-1, it appears that there are some addictive interactions (Figure 7b). However, adding metformin to linsitinib does not seem to be a good strategy since OC cells showed better responsiveness to linsitinib alone. Nonetheless, linsitinib seems to be a good drug for blocking the IGF-1 pathway.

## 3. Discussion

The microenvironment of malignant ascitic fluid (MAF) is rich in several proteins that support tumor cell growth, progression, and metastatic outgrowth [36,37], such as high expression levels of IGF receptors [38]. In this study, we showed that IGF-1 is overexpressed in high-grade serous carcinoma (HGSC) cell lines, specifically OVCAR3 cells, at both mRNA and protein levels, being a good cell line model to study the IGF-1 inhibition via knockdown of this gene by siRNA or IGF-1R inhibition with linsitinib. Our data prove that the blockade of the IGF-1 signaling pathway in OC cell lines decreases its cellular viability, and that combined linsitinib (inhibitor of IGF-1R) and metformin have an antagonistic effect, resulting in a less anti-neoplastic effect on OC cell lines.

The blockade of the IGF signaling pathway results in the inhibition of the PI3K/Akt and ERK1/2 pathways [27], leading to reduced cell growth and proliferation, decreased protein and fatty acid synthesis, and a diminished paracrine and endocrine release of pro-proliferative factors [28]. Our results support these previous findings by the decreased cellular viability of OVCAR3 and OVCAR3 siIGF-1 cells following linsitinib treatment. However, these results show that combining metformin with linsitinib does not produce a synergistic effect; instead, an antagonistic interaction is observed.

It is well established that metformin activates AMPK, inhibiting cell growth, mediated via the PI3K/Akt pathway. Previous research, such as that by Cantrell et al. and Xie et al., demonstrated that metformin inhibits cancer cell proliferation and promotes progesterone receptor expression in endometrial cancer, effects partially mediated through inhibition of the mTOR pathway [39,40]. Normal cell growth is maintained by a balance between the AMPK/Akt and mTOR pathways [39,41], and its deregulation can lead to metabolic disorders, resistance to apoptosis, and increased proliferation [41,42]. Under typical conditions, receptor tyrosine kinases regulate these pathways, especially the IGF-1R pathway [43]. Interestingly, recent suggestions indicate that mTOR inhibition might induce a feedback mechanism that paradoxically activates IGF-1 signaling, potentially reducing the efficacy of mTOR inhibitors [44].

Metformin has been associated with a decreased risk of OC in diabetic patients, increased progression-free survival, and overall survival in OC patients [45,46,47]. These benefits might result from metformin’s ability to regulate a disturbed energy balance, modulate IGF-1 pathway activity, and reduce chronic inflammation, which is commonly observed in diabetic and oncological patients [48]. Elevated adiposity and altered IGF-1 levels are also linked with OC, where insulin’s tumor-enhancing effects often activate the PI3K/Akt-mTOR pathway, a key regulator of cell growth, proliferation, and survival [49,50,51,52,53,54,55]. Metformin’s efficacy in reducing IGF-1 levels and tumor burden is particularly notable in patients on a high-energy diet, suggesting a greater effect in metabolically disrupted environments, which could be the reason why we did not reach the IC_50_ on cell lines, since the culture medium of the cells has glucose. The PI3K/Akt-mTOR pathway, frequently upregulated in OC, plays a crucial role in IGF-1 signaling [56,57,58], promoting cell cycle progression and survival and inhibiting apoptosis [57,59,60]. Within this pathway, mTOR functions as a pivotal regulated by Akt and inhibited by AMPK, a serine/threonine protein kinase that acts as a sensor of cellular energy status and is influenced by adenosine monophosphate (AMP)/adenosine triphosphate (ATP) levels [61]. Recent studies suggest that AMPK, beyond its role as an energy sensor, also regulates cell proliferation, growth, and autophagy [62]. For example, a caloric restriction diet, which increases AMPK activation while decreasing Akt and mTOR activation, mirrors the effects observed in high-energy diet groups treated with metformin. Under nutrient-deprived conditions that lead to energy depletion, AMPK activation transmits stress signals to mTOR through intermediates like tuberous sclerosis 2 and raptor, ultimately inhibiting mTOR activity [63].

In summary, modulating dietary intake can influence host metabolism and the tumor microenvironment, significantly affecting OC progression. A restricted diet is strongly linked to slowing cancer progression through mechanisms involving the IGF-1 pathway and mTOR activation [64,65]. The mTOR pathway is involved in coordinating cellular senescence processes [66], and its inhibition by rapamycin or similar compounds has been shown to prevent cancer in various models [67]. However, mTOR-driven senescence can also confer a selective survival advantage on cancer cells [64]. The complex relationship between senescence and cancer is highlighted by the fact that cellular senescence can block tumorigenesis [68], and it also involves multiple pathways that metformin can modify, resulting in decreased tumor formation.

**Figure 8:**
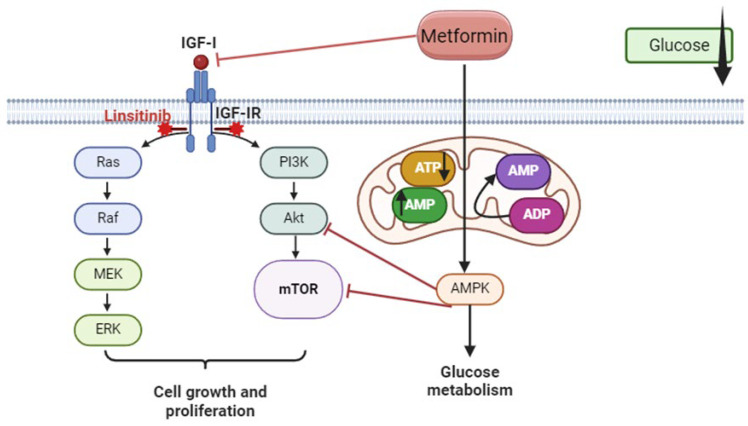
Schematic representation of metformin’s possible interaction with linsitinib. The most common pathway involves the activation of AMPK, which regulates energy metabolism by modulating complex 1 of the respiratory chain in mitochondria by changes in the AMP/ATP ratio, which inhibits Akt and mTOR. Metformin binds with IGF-1 and modulates pathways involved in tumor progression. Upon binding, metformin inhibits the PI3K/Akt/mTOR and Ras/Raf/ERK pathways, leading to reductions in cell proliferation, thereby causing tumor cell death. Metformin, through AMPK activation and mTOR inhibition, could increase glucose uptake and glycolysis and have better efficiency in low-glucose media. The arrows ↑ ↓ indicate upregulation and downregulation, respectively. The drug linsitinib blocks IGF-1R (represented by the red *), which helps to block the IGF-1 signaling pathway. ADP—adenosine diphosphate; Akt—Ak strain transforming; AMP—adenosine monophosphate; AMPK—adenosine monophosphate-activated protein kinase; ATP—adenosine triphosphate; Erk—extracellular-signal-regulated kinase; IGF-I—insulin-like growth factor 1; IGF-IR—insulin-like growth factor 1 receptor; MEK—mitogen-activated protein kinase; mTOR—mammalian target of rapamycin; PI3K—phosphatidylinositol 3-kinase; Raf—rapidly accelerated fibrosarcoma; Ras—rat sarcoma. Figure created in BioRender.com.

Our findings suggest that the antagonistic effect observed when combining metformin and linsitinib might be due to the presence of high glucose levels (Figure 8). High glucose appears to protect against metformin’s cytotoxic effects by sustaining glycolytic metabolism, thus maintaining cellular ATP levels even when metformin inhibits mitochondrial oxidative metabolism. Metformin accumulates in the mitochondrial matrix in the presence of a polarized mitochondrial membrane potential. It reversibly inhibits mitochondrial complex I of the respiratory chain, thereby repressing the coupling of redox and proton transfer domains and suppressing ATP production [69,70,71,72,73]. Another study indicates that metformin’s dose-dependent inhibition of cancer cell proliferation may involve a metabolic shift to glycolysis due to the suppression of mitochondrial complex I, limiting the movement of glucose- and glutamine-derived intermediates into the tricarboxylic acid (TCA) cycle and reducing acetyl-CoA levels necessary for lipid biosynthesis [74,75]. Cancer cells have a higher demand for ATP than normal cells, which has vital consequences under metformin treatment. As a result, glucose concentrations in cancer are often 3 to 10 times lower than in normal tissues [76]. Cancer cell lines with impaired glucose utilization in low-glucose media are more sensitive to metformin. Thus, glucose concentration is a critical factor in determining cancer cell sensitivity to metformin [75].

Summing up, the results presented in this study suggest that although both drugs target the same signaling pathway, high glucose levels may mitigate metformin’s cytotoxicity by providing a fuel source for glycolytic metabolism. This metabolic support allows for the maintenance of ATP levels, even when metformin blocks mitochondrial oxidative metabolism. Enhanced glycolytic metabolism induced by metformin requires AMPK activation, and the availability of glucose enables glycolytic processes to function efficiently [77]. In glucose-limited conditions, insufficient AMPK activation by metformin leads to a lack of fuel for glycolytic metabolism. Moreover, mTOR signaling is inhibited in an AMPK-independent manner, exacerbating metabolic deficiencies and leading to ATP depletion, energy collapse, and cell death [78]. Interestingly, non-cancer cells are not sensitized to metformin under glucose deprivation, likely due to their ability to utilize alternative substrates to maintain ATP levels [77].

While our results are promising, there are some points that warrant further investigation, such as the effect of the combination of linsitinib and metformin under glucose-limited conditions in cancer cell lines. Also, it would be valuable to analyze the impact of blocking the IGF-1 pathway on downstream components such as mTOR and AMPK to determine whether their mRNA and protein expression levels increase or decrease.

## 4. Materials and Methods

### 4.1. Cell Lines and Culture Conditions

The non-tumoral ovarian cell line (HOSE 6.3) was established from a normal ovary, surgically removed from patients with non-malignant disease [79]. The OC cell line (OVCAR3) was established in 1983 by Hamilton et al. from the malignant ascitic fluid (MAF) of a patient with high-grade serous carcinoma (HGSC) after a combination of chemotherapy with cyclophosphamide, adriamycin, and cisplatin [80]. OVCAR8 was selected as an HGSC model, particularly since it is described as a carboplatin-resistant OC cell line retrieved from an HGSC patient after a high-dose carboplatin treatment [81]. The cell lines (HOSE 6.3, OVCAR3, and OVCAR8) were kindly provided by Doctor Francis Jacob, Gynecological Cancer Center and Ovarian Cancer Research, Department of Biomedicine, University Hospital Basel and University of Basel, Basel, Switzerland. OVCAR8 PTX R P was previously established in our laboratory from parental OVCAR8 by pulse exposure [82]. All the characteristics of the cell lines used are described in Supplementary Data, Table S1. Cells were grown in a complete RPMI 1640 medium, which contained L-Glutamine, 25 mM HEPES, and 2.2 g/L of NaHCO_3_ (PAN-Biotech, catalog number P04-22100, Aidenbach, Germany). The medium was supplemented with 10% (*v*/*v*) inactivated and filtered fetal bovine serum (FBS; Biowest, catalog number S181A, Nuaillé, France) and 1% (*v*/*v*) penicillin/streptomycin (ThermoFisher Scientific, catalog number 15140122, Waltham, MA, USA). All cell lines were maintained at 37 °C and 5% CO_2_, with a humidified atmosphere. The experiments were performed when cells grew exponentially and presented less than 90% confluency. All cell lines were authenticated using short tandem repeat profiling and were regularly tested for the absence of mycoplasma using PCR.

### 4.2. RNA Isolation, cDNA Synthesis, and Quantitative Real-Time PCR

RNA isolation for quantitative real-time PCR was performed using the TRI Reagent (Sigma-Aldrich, catalog number T9424, Molecular Research Center, Inc., Saint Louis, MO, USA), according to the manufacturer’s instructions. The quantification of RNA was achieved by spectrophotometry (NanoDrop 1000 Spectrophotometer V3.8, ThermoFisher Scientific, Waltham, MA, USA). cDNA was synthesized with the SuperScript IV Reverse Transcriptase (Invitrogen, catalog number 18090010, ThermoFisher Scientific) according to the supplier’s instructions. The iQ SYBR Green Supermix Kit (Bio-Rad Laboratories, Inc., catalog number 1708880, Lisboa, Portugal) was used for amplification on the iQ Thermal Cycler (Bio-Rad) coupled to CFX Manager Software (version 1.0, Bio-Rad), as follows: initial denaturing step at 95.0 °C for 3 min; 37 cycles at 94.0 °C for 20 s; 61.0 °C for 30 s; and 72.0 °C for 30 s. Temperatures from 65.0 to 95.0 °C, with increments of 0.5 °C for 5 s, were included in the melt curves. Primers were used at a final concentration of 100 nM. Primers for *IGF-1* were as follows: forward: 5′-AAGCAATGGGAAAAATCAGCAGT-3′ and reverse: 5′-CAGAGCTGGTGAAGGTGAGC-3′; GAPDH: forward: 5′-ACAGTCCAGCCGCATCTTC-3′ and reverse: 5′-GCCCAATACGACCAAATCC-3′; and β-Actin: forward: 5′-AATCTGGCACCACACCTTCTA-3′ and reverse 5′-ATAGCACAGCCTGGATAGCAA-3′. For each data point, triplicated experiments were performed. The results were normalized against the housekeeping genes GAPDH and actin expression levels and analyzed through the ΔΔCT method. Overexpression of a gene was determined based on an mRNA-level fold change ≥ 1 relative to that of normal cells.

### 4.3. Protein Extracts and Western Blotting

For total protein extracts, cells were harvested by centrifugation and resuspended in lysis buffer (50 mM Tris pH 7.5; 150 mM NaCl; 1 mM EDTA; 1% Triton-100) containing a protease inhibitor cocktail (Sigma-Aldrich, catalog number 11836170001). Protein quantification was performed using a Pierce™ BCA Protein Assay Kit (ThermoFisher Scientific, catalog number A55864, Waltham, MA, USA), according to the manufacturer’s instructions. A total of 40 μg of protein lysate was resuspended in SDS-sample buffer (375 mM Tris pH 6.8; 12% SDS; 60% glycerol; 0.12% bromophenol blue; 600 nM DTT), boiled for 3 min at 100 °C, and proteins were separated on a 12% SDS–PAGE gel. After electrophoresis, proteins were transferred to a nitrocellulose membrane (Amersham) using the Trans-Blot Turbo Transfer System (Bio-Rad, Laboratories, Inc., Hercules, CA, USA). The membrane was blocked with 5% non-fat dried milk in TBST (50 mM Tris pH 7.5; 150 mM NaCl, 0.05% Tween-20) for 1 h at room temperature (RT) with mild agitation. Primary antibodies were diluted in 1% non-fat dried milk, as follows: mouse anti-IGF-1 (dilution: 1:100, sc-74116 clone W18, Santa Cruz Biotechnology, Heidelberg, Germany) and mouse anti-α-tubulin (1:5000, T568 Clone B-5-1-2, Sigma-Aldrich). After washing in TBST, the membrane was probed for 1 h at RT with horseradish peroxidase (HRP)-conjugated secondary antibodies, diluted at 1:1500 (anti-mouse, Vector). Proteins were visualized using the Enhanced Chemiluminescence (ECL) method with a ChemiDOc (Bio-Rad), and the relative signal intensity of the bands was determined as normalized against α-tubulin intensity levels using Image Lab 6.1v software.

### 4.4. siRNA Transfection

HOSE6.3 and OVCAR3 cells (origin described in Section 4.1) were seeded in 6-well plates at a density of 4 × 10^5^ and 5 × 10^5^, respectively, and incubated for 24 h in complete media to allow the cells to adhere. Twenty-four hours later, the medium was replaced with fresh medium, and the cells were transfected using INTERFERin siRNA Transfection Reagent (PolyPlus, New York, NY, USA) following the manufacturer’s instructions. Transfection was performed using 10 µM of a validated siRNA sequence against IGF-1 (siIGF-1) (Santa Cruz Biotechnology Inc., catalog number sc-37193) or 20 µM of a validated negative control siRNA (siNEG.) (AllStars Negative Control siRNA, catalog number 1027281, Qiagen, Germantown, MD, USA). After 48 h, RNA and protein were extracted as described above.

### 4.5. Drugs

Metformin and linsitinib were purchased from Selleckchem (Houston, TX, USA) and dissolved in distilled water and dimethyl sulfoxide (DMSO; AppliChem, catalog number A3672,0050, Barcelona, Spain), respectively, and then stored at −80 °C (final concentrations of 1 M metformin and 150 mM linsitinib), according to the manufacturer’s instructions. Immediately before use, an aliquot was diluted in a culture medium to the desired concentrations.

### 4.6. Drug Treatment

The IC_50_ values were first obtained for each drug alone for OVCAR3 and HOSE6.3 cell lines. The IC_50_ for all drugs was achieved by comparing treated cells with control cells (considered 100% viable) containing 1% (*v*/*v*) of the vehicle (DMSO or distilled water). For the single drug treatment, cells were treated for 48 h with metformin (80 to 10,000 μM) [35] and linsitinib (0.7 to 100 μM) [83]. Combination studies were performed according to the previously described method [35], using increasing concentrations of both drugs in a fixed ratio, as suggested by Chou–Talalay [84]. Linsitinib was combined in a simultaneous treatment with metformin in a fixed-dose ratio of 48 h. We used five combinations of drugs: 8.75 μM (0.25 × IC_50_) of linsitinib and 31.25 μM of metformin; 17.5 μM (0.5 × IC_50_) of linsitinib and 62.5 μM of metformin; 35 μM (IC_50_) of linsitinib and 125 μM of metformin; 70 μM (2 × IC_50_) of linsitinib and 250 μM of metformin; and 140 μM (4 × IC_50_) of linsitinib and 500 μM of metformin.

### 4.7. Cell Viability Assay

To determine the effect of drug treatments on cellular viability, a resazurin-based assay—Presto Blue (PB)—was performed. Briefly, 7.5 × 10^3^ cells/well of HOSE6.3 and 10 × 10^3^ cells/well of OVCAR3 were seeded into a 96-well plate in complete media, incubated at 37 °C and 5% CO_2_, and allowed to adhere overnight. After 24 h, cells were exposed to increasing concentrations of drugs and incubated under the same conditions. After 48 h, the culture medium was removed, and 50 μL/well of PrestoBlue^TM^ Cell Viability Reagent 1× (ThermoFisher Scientific, catalog number A13262) was added. Cells were incubated for 1 h, protected from light, at 37 °C and 5% CO_2_. Fluorescence was measured (560 nm excitation/590 nm emission) using a Bio Tek Synergy^TM^ 2 multi-mode microplate reader (BioTek, Winooski, VT, USA).

### 4.8. Drug Interaction Analysis

To measure drug interaction between linsitinib and metformin, we estimated the expected drug combination responses based on the zero-interaction potency (ZIP), Bliss Independence, Loewe, and High Single Agent (HSA) reference models using SynergyFinder 2.0 Software [34]. Positive and negative synergy scores denote synergy and antagonism, respectively. The cNMF algorithm implemented in SynergyFinder 2.0 was used for the estimation of outlier measurements [85].

### 4.9. Apoptosis Detection Using Annexin V/PI Double Staining

For apoptosis detection, after 48 h of treatment with linsitinib (35 µM), metformin (500 µM), or a combination of both (35 µM of linsitinib and 500 µM of metformin), floating and adherent OVCAR3 and OVCAR3 siIGF-1 cells were collected and processed using the Annexin V-FITC Apoptosis Detection Kit (eBioscience, catalog number 88-8005-74, Vienna, Austria) according to the manufacturer’s instructions. Fluorescence was assessed by the BD Accuri™ C6 Plus Flow cytometer (BD Biosciences, Qume Drive, San Jose, CA, USA), and data were analyzed with BD Accuri^TM^ C6 Plus software, version 1.0.27.1 (www.bdbiosciences.com). For these analyses, at least 20,000 events per sample were collected.

### 4.10. Statistical Analysis

All assays were carried out in triplicate with at least three independent experiments. Data are expressed as the mean ± standard error of mean deviation (SEM). Statistical analysis was carried out in GraphPad Prism Software Inc. v9 (GraphPad Software Inc., San Diego, CA, USA) using ordinary one-way or two-way ANOVA followed by Tukey’s or Šídák’s multiple comparison test, and values of * < 0.05, ** < 0.01, *** < 0.001, and **** < 0.0001 were considered statistically significant.

## 5. Conclusions

Linsitinib inhibition of the IGF-1 signaling pathway is a promising therapeutic option to treat OC patients, demonstrating significant anti-cancer effects in in vitro assays. However, it is crucial to identify drugs that could enhance or impair its therapeutic efficacy.

The findings presented in this study indicate that the combination of linsitinib and metformin has an antagonistic effect in OVCAR3 cells, resulting in a reduced anti-neoplastic efficacy when compared with linsitinib monotherapy. Beyond its effect on PI3K/Akt/mTOR and Ras/Raf/ERK pathways, linsitinib modulates ABC transporter-mediated multidrug resistance, increasing the accumulation of substrate anti-cancer drugs inside the cells. Therefore, future works exploring the combination of paclitaxel (a substrate of ABC transporter) and linsitinib would be valuable in the context of OC.

Metformin and linsitinib, through their multifaceted mechanisms of action, provide a strong rationale for their use in OC treatment. Ongoing and future clinical trials will be crucial in determining the optimal use of these agents, individually and in combination with a restricted diet, to improve the prognosis and survival of OC patients.

## Data Availability

The data presented in this study are available in this article and Supplementary Material.

## Supplementary Materials

The following supporting information can be downloaded at https://www.mdpi.com/article/10.3390/ijms252211935/s1.

## Abbreviations

Akt	Ak strain transforming
AMPK	Monophosphate-activated protein kinase
Bcl	B-cell lymphoma
CCC	Clear-cell carcinoma
EC	Endometrioid carcinoma
ERK	Extracellular-signal-regulated kinase
HGSC	High-grade serous carcinoma
HSA	High single agent
IGF	Insulin growth factor
IGF-1	Insulin-like growth factor 1
IGF-1R	Insulin-like growth factor 1 receptor
IGFBP1	Insulin-like growth factor binding protein 1
IR	Insulin receptor
JAK1/2	Janus kinases 1 and 2
LGSC	Low-grade serous carcinoma
MAP	Mitogen-activated protein
mTOR	Mammalian target of rapamycin
OC	Ovarian cancer
PARP	Poly ADP ribose polymerase
PI3K	Phosphatidylinositol 3-kinase
RAF	Rapidly accelerated fibrosarcoma
siIGF-1	siRNA of IGF-1
siNEG	siRNA of negative control
STAT3	Signal transducer and activator of transcription 3
VEGFR	Vascular endothelial growth factor receptor
ZIP	Zero interaction potency
