# Supplementary material for: Metformin Impairs Linsitinib Anti-Tumor Effect on Ovarian Cancer Cell Lines
Source: Int J Mol Sci. 2024 Nov 6;25(22):11935. doi: 10.3390/ijms252211935 (PMC11594113; doi:10.3390/ijms252211935)
Supplement: Supplementary file 1 [file ijms-25-11935-s001.zip › ijms-3289488-supplementary.pdf]

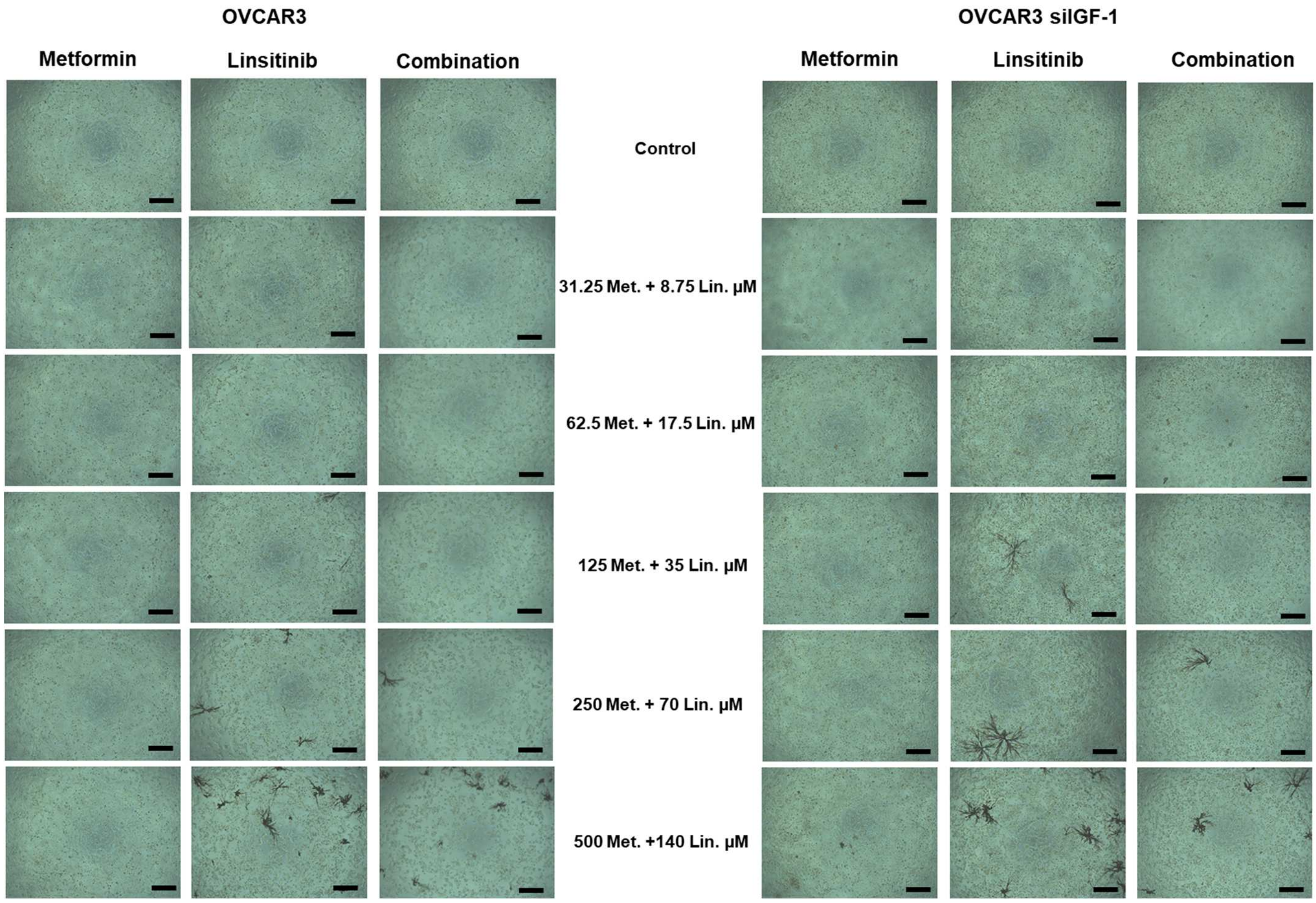

Figure S1. Morphological characterization of OVCAR3 and OVCAR3 siIGF-1 cells after drug treatment. Microscopy images of OVCAR3 and OVCAR3 siIGF-1 cells after exposure to the vehicle, linsitinib, metformin, and a combination of linsitinib and metformin for 48 h. All assays were performed in triplicate in at least three independent experiments. Magnification 50x and scale bar represents 200 µm.

Table S1. Information on ovarian cancer cell lines retrieved from Cellosaurus portal.

Name	Category	Origin	Diagnosis	Treatment (before generation of cell line)	Reference
HOSE6.3	Normal-like cell line	Epithelial cell of ovary	Human Ovarian Surface Epithelial	Not described	https://www.cellosaurus.org/CVCL_7673
OVCAR3	Cancer cell line	Ovarian cancer (Ascites)	High-grade ovarian serous adenocarcinoma	Chemotherapy (cyclophosphamide, adriamycin and cisplatin	https://www.cellosaurus.org/CVCL_0465
OVCAR8	Cancer cell line	Ovarian adenocarcinoma	High-grade ovarian serous adenocarcinoma	Chemotherapy (carboplatin)	https://www.cellosaurus.org/CVCL_1629
